# The role of blood urea nitrogen to serum albumin ratio in the prediction of severity and 30‐day mortality in patients with COVID‐19

**DOI:** 10.1002/hsr2.606

**Published:** 2022-05-06

**Authors:** Toktam Alirezaei, Saeede Hooshmand, Rana Irilouzadian, Behzad Hajimoradi, Sadra Montazeri, Arash Shayegh

**Affiliations:** ^1^ Men's Health and Reproductive Health Research Center Shahid Beheshti University of Medical Sciences Tehran Iran; ^2^ Department of Cardiology, School of Medicine Shahid Beheshti University of Medical Sciences Tehran Iran; ^3^ School of Medicine Shahid Beheshti University of Medical Sciences Tehran Iran

**Keywords:** blood urea nitrogen, COVID‐19, mortality, serum albumin, severity of disease

## Abstract

**Background:**

Considering the role of higher blood urea nitrogen and lower serum albumin (SA) levels in deceased coronavirus disease 2019 (COVID‐19) patients, an increased blood urea nitrogen to SA (B/A) ratio may help to determine those at higher risk of critical illness. This study aimed to evaluate the correlation of the B/A ratio with severity and 30‐day mortality in COVID‐19 patients.

**Methods:**

A total of 433 adult patients with COVID‐19 were enrolled. The laboratory markers were measured on admission. Disease severity was categorized into mild disease, severe pneumonia, acute respiratory distress syndrome (ARDS), sepsis, and septic shock. The mortality was followed for 30 days after admission. *χ* ^2^ test, Fisher's exact test, and Mann–Whitney *U* test were performed, as appropriate. Also, logistic regression and the receiver operating characteristic (ROC) curve for the B/A ratio are included.

**Results:**

Thirty‐day mortality rate was 27.25%. The frequency of mild, severe pneumonia, ARDS, sepsis, and septic shock was 30.72%, 36.95%, 24.02%, 6.00%, and 2.31%, respectively. B/A ratio and SA levels were statistically different between alive and deceased patients. The mean B/A ratio was different among classified disease severities, except for mild disease. Logistic regression revealed the B/A ratio as an independent risk factor for sepsis after adjusting for age and sex. ROC analysis showed B/A ratio had an area under the curve (AUC) of 0.733 for mortality at the cutpoint of 4.944. AUC for sepsis was 0.617 which was greater than other disease severities.

**Conclusion:**

The results showed that B/A ratio and SA levels are associated with mortality of COVID‐19 patients. A higher B/A ratio is, additionally, associated with COVID‐19 severity, except in mild cases and it can act as an independent risk factor in sepsis. However, a greater B/A ratio is not a significant predictor of COVID‐19 severity, but it can predict mortality. Therefore, we suggest this marker for clinical assessment of patients with severe COVID‐19.

## 1 INTRODUCTION

Coronavirus disease 2019 (COVID‐19), also known as the 2019 novel coronavirus, is the most critical issue around the world today.^1^ It has started in Wuhan, China, in December 2019 and spread around the world quickly by human‐to‐human transmission, and is announced as a pandemic respiratory disease by the World Health Organization (WHO) in March 2020. COVID‐19 is infecting more patients each day with worldwide mortality exceeding 4 million to date (August 2021).^2^, ^3^, ^4^, ^5^ The main site of infection in COVID‐19 is the respiratory system and the main clinical presentations include fever, cough, breathlessness, and pneumonia. Severe cases with acute respiratory distress syndrome (ARDS) require intensive care unit (ICU) admission and mechanical ventilation. Multiple organ failure may occur in some cases of COVID‐19 and result in death.^1^ In contrast, some may remain asymptomatic and act as carriers.^6^ In addition, COVID‐19 may have a long incubation period.^7^ Accordingly, it is important to predict the disease severity and clinical progression of COVID‐19.^8^, ^9^

It has been suggested that COVID‐19 enters the body cells through binding to angiotensin‐converting enzyme‐2 (ACE2) receptor,^10^ which mainly helps in blood pressure regulation. This type I membrane protein is expressed in endothelium and body organs including lungs, heart, kidneys, and intestines at varying levels,^11^ which renders these tissues more vulnerable to damage by COVID‐19.^12^, ^13^ It has been shown that serum albumin (SA) levels result in downregulation of ACE2.^14^ Additionally, lower SA concentrations were seen in severe forms of COVID‐19 in comparison with less severe forms.^15^ Other studies have also emphasized the significance of measuring this parameter in patients with COVID‐19.^16^ Therefore, the study of its predictive value for severity and mortality in COVID‐19 is of great importance.

Blood urea nitrogen (BUN), the end product of nitrogen metabolism, has been previously suggested as a useful predictor of cardiovascular morbidity and mortality,^17^ as well as mortality in patients with H1N1 pneumonia.^18^ In terms of COVID‐19, elevated baseline BUN levels were associated with severe COVID‐19 and adverse outcomes.^19^ On the other hand, the B/A ratio had shown a high predictive value for in‐hospital mortality in COVID‐19 patients.^20^ Therefore, we hypothesized that the combination of BUN and SA levels (BUN to SA [B/A] ratio), can be a valuable predictor of COVID‐19 severity, as well. As far as we are concerned, the predictive value of the B/A ratio for COVID‐19 severity categorized by WHO classification has not been studied, previously. Hence, the present study aimed to evaluate SA level and B/A ratio for the prediction of COVID‐19 severity and 30**‐**day mortality in patients admitted to the hospital with COVID‐19.

## 2 MATERIALS AND METHODS

### 2.1 Study design

**FIGURE 1:**
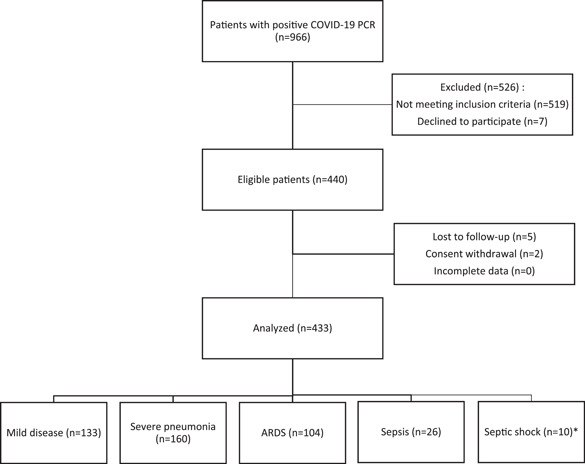
Flowchart of the studied group. *Due to an inadequate number of patients in the septic shock group, further in the study, these patients were merged into the sepsis group. ARDS, acute respiratory distress syndrome; COVID‐19, coronavirus disease 2019; PCR, polymerase chain reaction

In this descriptive cross‐sectional study, adult patients (above the age of 18 years), diagnosed with COVID‐19 based on positive polymerase chain reaction results, admitted to Shohada‐e‐Tajrish Hospital, a referral medical center in Tehran, Iran, from February to May 2020 were included. Cases with the moderate‐to‐severe disease were hospitalized. Patients with a positive history of hospitalization in the past 90 days, chronic immunodeficiency (such as patients with human immunodeficiency virus, receiving chemotherapy or those who used prednisolone or other immunosuppressive agents), advanced liver disease, receiving dialysis, or patients with chronic renal disease (serum creatinine level >1.5 mg/dl), and patients with cancer or cachexia were not included into the study. As a result, 433 eligible patients were enrolled in the study consecutively after signing the written informed consent (Figure 1).

The demographic and clinical presentation of the patients were extracted from the hospital's medical records. The disease severity of COVID‐19 in the participants was categorized into mild disease, severe pneumonia, ARDS, sepsis, and septic shock, based on WHO categorization^21^ (Figure 1). Mild cases included patients with COVID‐19 with uncomplicated upper airway infection, presenting with fever, fatigue, cough (with or without sputum), anorexia, myalgia, sore throat, dyspnea, nasal congestion, headache, and rarely diarrhea and nausea/vomiting. Severe cases were defined as COVID‐19 cases with severe pneumonia, presenting with fever or respiratory infection with a respiratory rate ≥30/min or SpO_2_ ≤ 93% at room air.

One blood sample was taken from all patients within 4 h of their admission in a standing position, collected in plain tubes, and sent to the laboratory within 2 h after collection. In the lab, the samples were kept at 2–8°C. For testing, the serum/plasma was separated from the samples by centrifuge and evaluated for the levels of BUN, albumin, and other electrolytes. BUN was measured using the enzymatic method with urease and glutamate dehydrogenase and SA level using Bromocresol green method and Latex coagulating nephelometric assay; other biochemical markers were measured using standard methods. The B/A ratio (mg/g) was calculated by dividing the BUN level (mg/l) by the albumin level (g/l).

The clinical progression and outcome of the disease in terms of survival or mortality were assessed 30 days after hospitalization by telephone calls and hospital records. In the case of posthospitalization death, the cause of death was asked from their first‐degree relative.

The protocol of the study was approved by the Ethics Committee of Shahid Beheshti University of Medical Sciences (code: IR.SBMU.RETECH.REC.1399.466). Any patient who was referred to another center or patients who did not respond to the 30‐day telephone call were considered lost to follow‐up and excluded from the study.

### 2.2 Statistical analysis

All statistical analyses were performed using the statistical software SPSS (version 25) and R (version 4.1.2). Two‐sided *p *< 0.05 were considered statistically significant. Categorical and continuous variables were presented as frequency, percentage, median, and interquartile range (IQR). The normality distribution of data was checked by the Kolmogorov–Smirnov test. Subsequently, the Mann–Whitney test was performed to compare age, duration of hospitalization, albumin, and B/A ratio between the subgroups of outcome variables. Associations between categorical variables were analyzed by the *χ* ^2^ test or Fisher's exact test. Multiple logistic regression was used to evaluate the association between each dichotomized disease severity and the B/A ratio. The Hosmer–Lemeshow test was applied to assess the goodness of fit for the logistic regression models. The area under the receiver operating characteristic (ROC) curve was used for the prediction of disease severity and in‐hospital mortality by B/A ratio.

## 3 RESULTS

Complete records of 433 COVID‐19 patients were available. The median (IQR) age was 60.38 (18.26) years and 60.74% were male (*n* = 263). The median (IQR) of the duration of hospitalization was 4.45 (5.70) days; 51 patients (11.78%) required intubation; 113 (26.1%) died during hospitalization and 5 others (1.15%) until 30 days after admission. There were 133 patients with mild disease (30.72%), 160 patients with severe pneumonia (36.95%), 104 patients with ARDS (24.02%), 26 patients with sepsis (6.00%), and 10 patients with septic shock (2.31%). Due to inadequate data in the septic shock group, the sepsis group and septic group were merged. The distribution of the variables among patients with different disease severities and between alive and deceased participants are shown in Table 1. As shown, the deceased patients had a greater male‐to‐female ratio (74/44), compared to the alive patients (189/126), higher mean age (72 vs. 56.0 years, respectively; *p* < 0.0001), and longer duration of hospitalization (4.0 vs. 2.0 days, respectively; *p* < 0.001). Among deceased patients, 43.2% (*n* = 51) required intubation, while none of the alive patients required intubation (*p* < 0.0001) (Table 1).

**TABLE 1:** Distribution of general and clinical characteristics according to disease severity and in‐hospital mortality

Variable	Outcome during hospitalization								Final outcome	
	Mild		Severe pneumonia		ARDS		Sepsis		Survival status	
	No	Yes	No	Yes	No	Yes	No	Yes	Alive	Died
Gender										
Male	185 (61.7)	78 (58.6)	164 (60.1)	99 (61.9)	197 (59.9)	66 (63.5)	243 (61.2)	20 (55.6)	189 (60.0)	74 (62.7)
Female	115 (38.3)	55 (41.4)	109 (39.9)	61 (38.1)	132 (40.1)	38 (36.5)	154 (38.8)	16 (44.4)	126 (40.0)	44 (37.3)
	*p* = 0.553		*p* = 0.711		*p* = 0.514		*p* = 0.506		*p* = 0.607	
Age (years)	63.5 (47.2–75.0)	59.5 (47.2–72.5)	64.5 (50.0–77.0)	56.0 (42.0–72.0)	57.0 (45.0–72.0)	72.0 (60.0–82.0)	62.0 (47.0–74.0)	57.0 (48.2–72.0)	56.0 (43.0–71.0)	72 (60.0–82.0)
	*p* = 0.155		*p* = 0.001		*p* < 0.0001		*p* = 0.593		*p* < 0.0001	
Hospital duration (days)	3.0 (1.0–6.7)	2.0 (2.0–3.0)	3.0 (1.0–5.0)	3.0 (1.0–6.0)	2.0 (1.0–4.0)	4.0 (1.0–7.0)	3.0 (1.0–5.0)	2.0 (1.0–7.0)	2.0 (1.0–4.0)	4.0 (1.0–8.0)
	*p* = 0.012		*p* = 0.663		*p* = 0.009		*p* = 0.549		*p* < 0.001	
Intubation										
No	252 (84.0)	130 (97.7)	243 (89.0)	139 (86.9)	298 (90.6)	84 (80.8)	353 (88.9)	29 (80.6)	315 (100.0)	67 (56.8)
Yes	48 (16.0)	3 (2.3)	30 (11.0)	21 (13.1)	31 (9.4)	20 (19.2)	44 (11.1)	7 (19.4)	0 (0.0)	51 (43.2)
	*p* < 0.0001		*p* = 0.506		*p* = 0.007		*p* = 0.171		*p* < 0.0001	
SA	4.2 (3.7–4.8)	4.2 (3.5–4.5)	4.2 (3.6–4.6)	4.2 (3.8–4.8)	4.2 (3.7–4.7)	4.3 (3.6–4.8)	4.2 (3.7–4.7)	4.1 (3.5–4.6)	4.3 (3.9–4.8)	3.9 (3.3–4.4)
	*p* = 0.111		*p* = 0.112		*p* = 0.670		*p* = 0.437		*p* < 0.0001	
B/A	4.2 (2.9–8.3)	4.1 (2.7–6.4)	4.5 (3.1–8.8)	3.8 (2.6–6.4)	4.0 (2.7–6.8)	5.0 (3.5–12.4)	4.1 (2.8–7.2)	6.3 (3.5–13.1)	3.6 (2.6–5.4)	8.8 (5.0–14.5)
	*p* = 0.108		*p* = 0.001		*p* < 0.0001		*p* = 0.019		*p* < 0.0001	

*Note*: Data were presented as median (IQR) and *n* (%). *p*‐values were calculated by *χ* ^2^ test, Fisher's exact test, and Mann–Whitney *U* test, as appropriate.

Abbreviations: ARDS, acute respiratory distress syndrome; B/A, blood urea nitrogen to albumin ratio; SA, serum albumin.

There was no difference in mean SA level among patients with different disease severities, while it was significantly lower in deceased patients compared to alive ones (3.9 vs. 4.3 g/dl, *p* < 0.0001). The mean B/A ratio was significantly higher in deceased patients, compared to alive ones (8.8 vs. 3.6; *p* < 0.0001) and also different among patients with different disease severities except for the mild one (Table 1).

Results of the logistic regression model for B/A ratio on the study outcomes, adjusted for age and sex, revealed B/A ratio as an independent risk factor for sepsis (adjusted odds ratio = 1.060, 95% confidence interval, CI [1.017, 1.104]). However, our results could not suggest the B/A ratio as a risk factor for mild disease, severe pneumonia, and ARDS. Hosmer–Lemeshow tests were performed to approve that the model fits well with the data (*p* > 0.05) (Table 2).

**TABLE 2:** Results of the logistic regression showing the relationship between B/A ratio and disease severity by adjusting for age and sex

Outcome variable	*β* (SE)	Adjusted odds ratio (95% CI)	*p*	Hosmer–Lemeshow test
Mild	−0.031 (0.019)	0.969 (0.934, 1.006)	0.101	$X^{2}=9.859$ *p *= 0.275
Severe pneumonia	−0.034 (0.018)	0.966 (0.932, 1.002)	0.064	$X^{2}=7.448$ *p *= 0.489
ARDS	0.029 (0.016)	1.029 (0.997, 1.063)	0.080	$X^{2}=6.443$ *p *= 0.598
Sepsis	0.058 (0.021)	1.060 (1.017, 1.104)	0.006	$X^{2}=11.489$ *p *= 0.176

Abbreviations: ARDS, acute respiratory distress syndrome; B/A, blood urea nitrogen to serum albumin; CI, confidence interval.

Hosmer‐Lemeshow tests were performed to approve that the model fits well with the data (*p* > 0.05).

**FIGURE 2:**
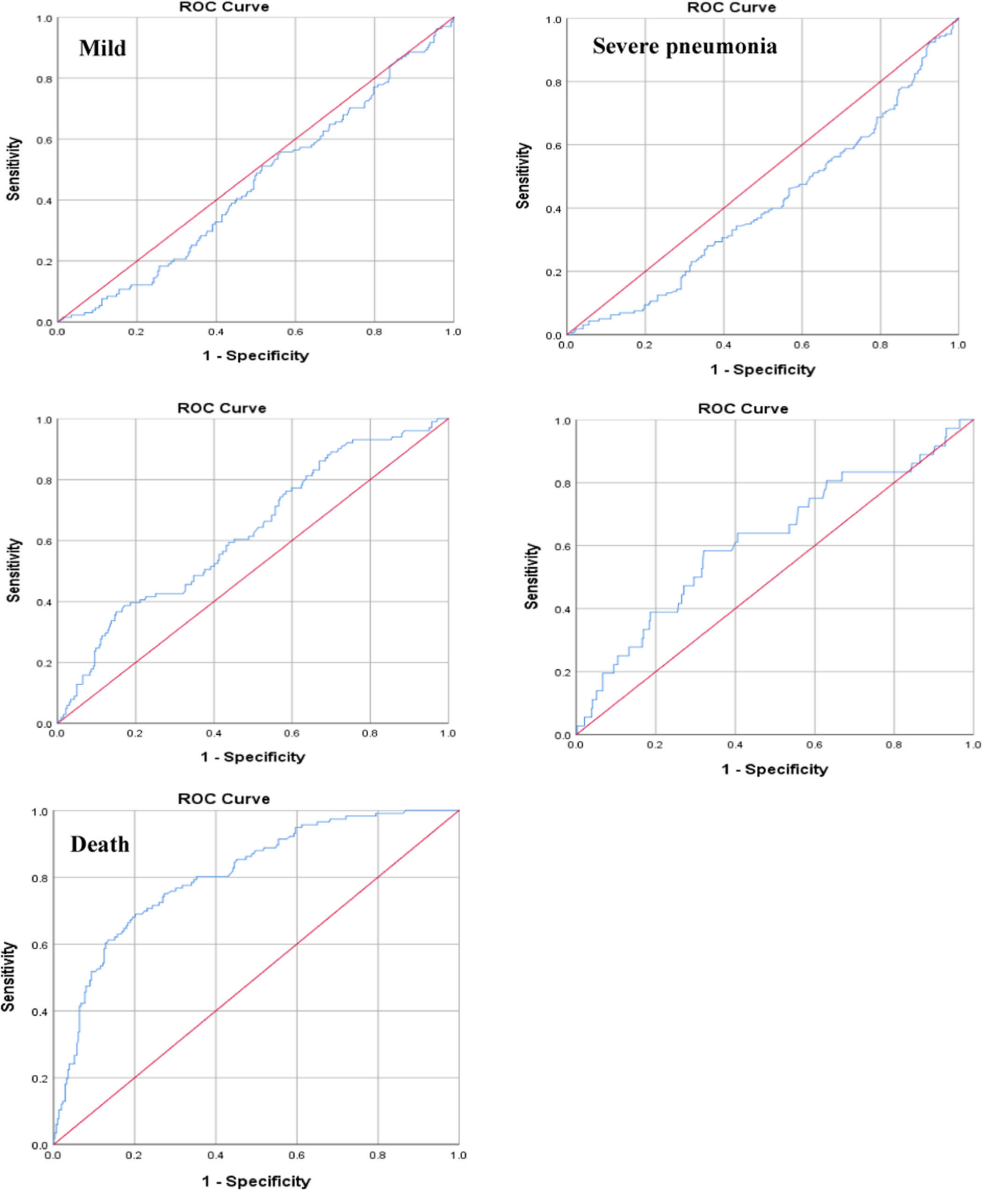
Receiver operator characteristic curves for prediction of disease severities and in‐hospital mortality by B/A ratio. B/A, blood urea nitrogen to serum albumin; ROC, receiver operating characteristic

Results of the ROC analysis showed B/A ratio had the area under the curve (AUC) value of 0.733 (95% CI [0.680, 0.787]) for mortality during hospitalization at the cutpoint of B/A ratio of 4.944 with 75.8% sensitivity and 70.8% specificity. AUC for sepsis was greater than other disease severities with AUC of 0.617 (95% CI [0.552, 0.712]), while 0.484 (95% CI [0.425, 0.544]) for mild disease, 0.441 (95% CI [0.385, 0.497]) for severe pneumonia and 0.558 (95% CI [0.494, 0.621]) for ARDS (Table 3) (Figure 2).

**TABLE 3:** Area under the curve and validity indices for prediction of disease severities and in‐hospital mortality by B/A ratio

Outcome variable	Cutpoint	AUC (95% CI)	Sensitivity (95% CI)	Specificity (95% CI)	PPV (95% CI)	NPV (95% CI)
Mild	3.737	0.484 (0.425, 0.544)	0.564 (0.475, 0.651)	0.404 (0.347, 0.462)	0.294 (0.239, 0.355)	0.677 (0.603, 0.746)
Severe pneumonia	3.954	0.441 (0.385, 0.497)	0.475 (0.395, 0.555)	0.406 (0.347, 0.468)	0.323 (0.264, 0.387)	0.564 (0.491, 0.635)
ARDS	4.038	0.558 (0.494, 0.621)	0.613 (0.511, 0.709)	0.501 (0.446, 0.557)	0.275 (0.218, 0.338)	0.807 (0.746, 0.859)
Sepsis	4.849	0.617 (0.552, 0.712)	0.638 (0.462, 0.791)	0.594 (0.543, 0.643)	0.126 (0.081, 0.183)	0.947 (0.911, 0.971)
Death during hospitalization	4.944	0.733 (0680, 0.787)	0.758 (0.670, 0.833)	0.708 (0.654, 0.758)	0.491 (0.416, 0.567)	0.887 (0.841, 0.923)

Abbreviations: ARDS, acute respiratory distress syndrome; AUC, area under curve; B/A, blood urea nitrogen to serum albumin; CI, confidence interval; NPV, negative predictive value; PPV, positive predictive value.

## 4 DISCUSSION

The results of the present study showed the findings of the 433 patients that were admitted to the hospital with the diagnosis of COVID‐19. The majority of the study population were male, in general, and in both deceased and alive patients, and the median age of deceased patients was higher. These results showed the importance of COVID‐19 in the elderly and male individuals, which is consistent with the results of previous studies. In a study by Goyal et al., in 393 patients that were admitted with confirmed COVID‐19, the median age was 62.2 years and 60.74% were male,^22^ which is similar to the results of our study. Others have also revealed that COVID‐19 incidence is more in older males with comorbidities.^23^

The results of the present study showed that the median duration of hospitalization was significantly longer in the deceased group (4.5 vs. 1.8 days) and none of the alive patients required intubation; a total of 118 died (27.2%). Categorizing patients according to WHO classification showed that the frequency of mild disease, severe pneumonia, ARDS, sepsis, and septic shock was 30.72%, 36.95%, 24.02%, 6.00%, and 2.31%, respectively. Therefore, most of our studied patients had mild to moderate disease severity. In a study on 16,000 patients in Tehran, Iran, the median duration of hospitalization was 5 days in deceased patients and 3 days in alive patients and the total case fatality rate (CFR) was 10.05%, higher in patients more than 65 years old (25.32%) and ICU patients (41.7%).^24^ The median duration of hospitalization reported in this study was similar to that of ours, while the CFR was much higher in our study, which can be due to the difference in the factors affecting the mortality of the patients, such as age, comorbidities, and disease severity.

Serum parameters have been suggested as an easy and available assessment tool for the prediction of COVID‐19 severity. The role of low SA levels (<3.5 g/dl) on COVID‐19 progression to respiratory failure and its significance as a predictor of COVID‐19 outcome, independent of age and comorbidities has been well described. The underlying mechanism of this association has been suggested to be related to the downregulation of ACE2 by SA, as well as the association of hypoalbuminemia with coagulopathy.^14^, ^15^, ^16^ ^,^ ^25^, ^26^, ^27^ A study by Kheir et al. on 181 COVID‐19 patients showed higher SA levels on admission were associated with a 72% decreased risk of developing venous thromboembolism for every 1 g/dl increase of SA level and a lower risk of developing ARDS, admission to ICU, and readmission to hospital within 90 days.^28^ The results of a meta‐analysis on 4659 patients also showed that nonsurvivors had significantly lower SA and higher BUN levels, suggesting these two factors as important predictors of mortality in patients with COVID‐19.^29^ This confirms the results in the present study that the deceased patients had lower SA levels. However, SA levels were not statistically different among COVID‐19 severities. A cohort study by Liu et al. conducted on 12,413 COVID‐19 patients, showed that among serum creatinine level, blood uric acid, and BUN, an elevated BUN level from baseline was associated with the highest risk of adverse outcomes and all‐cause mortality.^19^ Additionally, another study also showed BUN/creatinine ratio as an independent predictor of COVID‐19 severity and survival.^30^ The underlying mechanism of this association has been suggested to be related to the expression of ACE2 receptors in kidneys, which results in activating the renin–angiotensin–aldosterone system and increases the absorption of water and sodium in the kidney tubules, causing passive reabsorption of BUN, renal vasoconstriction, and worse prognosis in patients with underlying renal dysfunction.^31^ The results of a study by Cheng et al. revealed serum BUN ≥ 4.6 mmol/L as an independent predictor of in‐hospital mortality in patients with COVID‐19,^32^ which is in line with the results of the present study, although we considered SA levels and B/A ratio.

We hypothesized that the combination of BUN and SA levels (B/A ratio), previously indicated as an important predictor of progression of pneumonia into critical conditions^33^ and death,^34^, ^35^ can be a valuable predictor of COVID‐19 severity, as well. In a study performed by Kucukceran et al.,^20^ among 602 COVID‐19 patients, the BUN level and B/A ratio were significantly higher in deceased patients compared to alive patients. It also showed B/A ratio has a high predictive value for COVID‐19 in‐hospital mortality with an AUC value of 0.809 with the cutoff value of 3.9 mg/g.^20^ However, none have studied its value for the severity of COVID‐19 defined by WHO classification and our study was the first to evaluate this issue, as far as we are concerned. Our study demonstrated not only higher mean values of B/A ratio in deceased patients but also significant differences by different disease severities except the mild disease. The results of the present study suggested B/A ratio is an independent risk factor for sepsis in COVID‐19 patients. We also revealed that the B/A ratio did not have statistically significant predictive values for different COVID‐19 severities. However, it had a poor predictive value for in‐hospital mortality.

One of the limitations of the present study was the nonrandomized inclusion of patients in the study and in the groups. The nonrandomized categorization of the patients into groups was inevitable, as we had to categorize them according to the disease outcome. The study center is also a referral center, which reduces the bias of nonrandomized inclusion of patients in the study; thus, we included all patients in the study by the Census method. The second limitation is that we have measured the BUN and SA levels of participants once and did not consider the time‐dependent variations in these serum parameters. Another limitation of the study is the risk of bias in the results due to the effect of confounders on the results. So, we excluded the conditions that can affect the serum parameters to reduce the bias. Last but not least, this was a single‐center study and it is questionable whether the results of this study are replicable in other centers.

## 5 CONCLUSION

This study indicated that the B/A ratio and SA levels are statistically different between alive and deceased patients. B/A ratio is significantly greater among different COVID‐19 severities except for mild disease. We concluded that the B/A ratio was an independent risk factor for sepsis. However, a high B/A ratio is not a significant predictor of COVID–19 severity, but it can predict mortality. Considering the association of the B/A ratio in mortality and sepsis, and its ease of application, it is suggested to investigate this issue in larger populational studies, especially in patients with severe COVID–19.

## AUTHOR CONTRIBUTIONS

**Toktam Alirezaei**: Conceptualization; writing – original draft; and writing – review and editing. **Saeede Hooshmand**: Conceptualization; investigation; project administration; and resources. **Rana Irilouzadian**: Investigation; project administration; writing – original draft; and writing – review and editing. **Behzad Hajimoradi**: Conceptualization; project administration; supervision; and validation. **Sadra Montazeri**: Data curation; formal analysis; and resources. **Arash Shayegh**: Data curation; formal analysis; and methodology.

## CONFLICTS OF INTEREST

The authors declare no conflicts of interest.

## TRANSPARENCY STATEMENT

The manuscript is an honest, accurate, and transparent account of the study being reported; no important aspects of the study have been omitted; and any discrepancies from the study as planned (and, if relevant, registered) have been explained.

## ETHICS STATEMENT

All of the authors declare that this manuscript is not published elsewhere. Written informed consent was obtained from patients to publish this report in accordance with the journal's patient consent policy and all of the authors declare that the confidentiality of the patient was respected.

## Data Availability

The data that support the findings of this study are available on request from the corresponding author.
